# Sex differences in limitations of life-sustaining treatment in the intensive care unit: A nationwide cohort study

**DOI:** 10.1016/j.aicoj.2026.100190

**Published:** 2026-10-17

**Authors:** Elsa Hägglöf, Jesper Eriksson, Max Bell, Linn Hallqvist, Lars Engerström, Emma Larsson

**Affiliations:** a Department of Physiology and Pharmacology, Karolinska Institutet, Solna, Sweden; b Department of Perioperative Medicine and Intensive Care, Karolinska University Hospital, Stockholm, Sweden; c Department of Anaesthesia and Intensive Care Medicine, and Department of Biomedical and Clinical Sciences, Vrinnevi Hospital, Norrköping, Sweden; d Department of Cardiothoracic and Vascular Surgery and Anaesthesia, and Department of Medical and Health Sciences Linköping University Hospital, Linköping, Sweden

**Keywords:** Intensive care, Critical care, Gender, Sex, Life-sustaining treatment

## Abstract

**Background:**

Previous research suggests that limitations of life-sustaining treatment (LST) may be more common in women than men, but few studies have included data on clinical reasoning or limitation type. This study aimed to investigate sex differences in LST-limitations in the ICU and in their documented characteristics.

**Methods:**

All adult (≥18 years) ICU patients registered in the Swedish Intensive Care Register between 2019 and 2024 were included. A multivariable logistic regression model, adjusted for SAPS3 and age, analysed the association between sex and LST-limitations. Limitation characteristics were compared using Chi-square test (categorical variables) and Wilcoxon rank-sum test (numeric variables).

**Results:**

The cohort included 111,354 patients; 41.7% women. LST-limitations occurred in 24.4% of patients and were more frequent in women than in men (25.3% vs. 23.8%, p < 0.0001). In adjusted analyses women had higher odds of receiving an LST-limitation than men, both in the full cohort (OR 1.21, 95% CI 1.17−1.25) and among 30-day non-survivors (OR 1.24, 95% CI 1.18−1.32). Women received LST-limitations earlier than men (2.8 h after admission vs 4.5 h, p < 0.0001). Acute disease and therapy failure were more often documented reasons in men than in women (78.8% vs 74.3%, p < 0.0001; 21.2% vs. 18.6%, p = 0.0001), whereas autonomy and chronic disease were more frequent in women (7.8% vs 5.9%, p < 0.0001; 63.2% vs. 60.7%, p = 0.0021). Women were more often involved in decision-making (13.3% vs 11.8%, p = 0.0074).

**Conclusions:**

Women were more likely to receive LST-limitations and received them earlier. Men who died within 30 days were less likely to have had an LST-limitation than women. Documented reasons for LST-limitations differed by sex, and women had higher rates of documented patient involvement.

## Background

The intensive care unit (ICU) provides advanced treatment for the most critically ill patients with the potential to save lives. However, as the treatment can be distressing and even painful for the patient [1], deciding who will truly benefit from this level of care, and who will not, remains a crucial consideration. Decisions to limit life-sustaining treatment (LST) are common in the ICU and range from withdrawing or withholding individual interventions, such as dialysis, invasive ventilation, vasoactive drugs or cardiopulmonary resuscitation (CPR), to limiting all LSTs.

As LST-limitations in the ICU are both frequent and consequential, they must be based on relevant clinical variables and patient preferences. The frequency of these decisions varies between countries, and between hospitals within a country [2], but some common predictors of LST-limitations have been identified including age [3], illness severity measured by the Simplified Acute Physiology Score 3 (SAPS3) [4], frailty [5], chronic comorbidity and pre-admission functional status [6]. In addition to these established predictors, studies have suggested that women may be addressed with LST-limitations more often than men [7,8]. A higher proportion of LST-limitations is not inherently negative, since LST-limitations are important when intensive care risks causing more harm than good, but no evidence supports differing decision-making approaches for men and women.

Evidence regarding sex differences in LST-limitations is sparse, as most previous research has mainly observed a higher proportion of LST-limitations in women without providing data on clinical reasoning and the type of limitations. Interpretation has therefore remained cautious and further research has been requested [6,8]. We aimed to investigate sex differences in LST-limitations in the ICU and in the documented characteristics of these limitations.

## Methods

### Study design and population

We included all adult (≥18 years) patients treated in the ICU between 1 January 2019 and 15th August 2024 registered in the Swedish Intensive Care Register (SIR). For patients with multiple ICU admissions, only the first was included. Exclusion criteria were concealed or temporary personal identity number, emigration within 30 days after admission, missing SAPS3, patients from ICUs with more than 40% missing LST-decisions, and patients with missing data on LST-decisions. Missing LST-decision data was defined as complete absence of a reported treatment strategy in the registry. ICUs with >40% missing LST-decision data were excluded to minimise selection bias that could result from excluding individual cases; this threshold was pragmatically chosen and does not represent a standardised cutoff (the impact of alternative thresholds is shown in Supplementary Table 1).

### Covariates and outcome

SIR collects data from all 80 ICUs in Sweden and includes baseline demographics, comorbidities, variables from SAPS3, and details on treatments administered in the ICU. Following centralised validation by SIR, any incomplete or inconsistent entries, defined as values falling outside predefined limits, are returned to the respective ICUs for correction prior to inclusion in the final database.

Since 2016, SIR has included a quality indicator requiring documentation of a treatment strategy within 24 h of ICU admission. Detailed information became available in 2019 and therefore determined the study start. The treatment strategy specifies either no limitation or limitation of LST. LST-limitations involve withholding or withdrawing one or more predefined treatment categories recorded in the registry: invasive ventilation, non-invasive ventilation, dialysis, CPR, vasoactive drugs, or other treatments. No further information is available regarding treatments classified as “other treatments”. Each limitation can be assigned one or more of five predefined reasons: autonomy, acute disease, chronic disease, therapy failure, or other. Documentation also includes whether consultation occurred with the patient, next of kin, or another licensed healthcare professional. Under Swedish regulations, LST-limitations are decided by the patient-responsible physician, who is required to consult at least one other licensed healthcare professional before a decision to withhold or withdraw life-sustaining treatment is made. As only the treatment strategy is recommended to report to SIR, not all hospitals consistently report full details of LST-limitations. Moreover, full characteristics were unavailable for limitations recorded prior to ICU admission.

The primary outcome, LST-limitation, was defined as a documented decision to withhold or withdraw one or more LST. This included either a newly documented limitation during the ICU stay or a previously recorded limitation that was reaffirmed at the time of ICU admission. If multiple LST-limitations were documented during the ICU stay, only the first was considered for analysis. Secondary outcomes were characteristics of LST-limitations, including time to limitation, reason for limitation, type of limitation and consultation pattern. Time to LST-limitation was defined as the number of hours from ICU admission to documentation of the first LST-limitation. For LST-limitations recorded prior to ICU admission, time to LST-limitation was set to zero. Time from LST-limitation to death was defined as the number of days from documentation of the first LST-limitation to date of death.

SAPS3 was used to describe illness severity at the time of admission to the ICU. SAPS3 is a risk adjustment model used in SIR and globally to estimate mortality risk and evaluate intensive care nationally. Developed in collaboration with the European Society of Intensive Care Medicine in 2002, SAPS3 is composed of three boxes, where box 1 includes parameters before the initiation of intensive care, box 2 includes circumstances leading to the need for intensive care, and box 3 includes physiological disorders and their severity at admission [9]. Estimated mortality risk (EMR) was calculated from SAPS3 using the Swedish calibration which corresponds to estimated risk of 30-day mortality [10]. SAPS3 variables were also used to describe comorbidities and location before ICU admission. Comorbidity data included metastatic cancer, cancer therapies (e.g., chemotherapy, immunosuppression, radiotherapy, steroid treatments), chronic heart failure (New York Heart Association functional classification (NYHA) IV), hematologic cancer and cirrhosis. Reasons for ICU admission were extracted from SAPS3 and allowed for multiple predefined reasons, implying each patient could have several reasons.

All Swedish citizens are assigned a personal identity number at birth or upon immigration. The variable patient ‘sex’ was defined by ‘legal gender’, which is represented as a number within the identity number. Legal gender in Sweden is binary (male or female). Although it is possible to change one's legal gender in Sweden, it remains an uncommon occurrence and the variable primarily reflects sex assigned at birth. Therefore, the variable was referred to as sex in this study.

The identity number also enables linkage to national registries. SIR uses the personal identity number to continuously retrieve mortality data from the Swedish Population Register. Missing mortality data may result from concealed identity, temporary personal identity number, or emigration within 30 days of ICU admission.

### Statistical methods

Categorical variables were presented as numbers and percentages. Continuous variables without were presented as medians and interquartile ranges (IQR).

Differences between men and women in LST-limitations and characteristics such as timing, consultation pattern and limitation type were compared with chi-square tests for categorical variables and with Wilcoxon rank-sum tests for medians of continuous variables.

To investigate the association between sex and LST-limitations in the ICU logistic regression was used. Univariable regression was used to analyse the association between sex and LST-limitations and multivariable regression to analyse the association adjusted for age and SAPS3. Analyses were performed in the full cohort and in 30-day non-survivors. Odds ratios (OR) were presented with corresponding 95% confidence intervals (CI).

To assess the potential impact of the COVID-19 pandemic on LST decision-making, we conducted supplementary analyses stratified by pre-COVID-19 (before March 11, 2020), COVID-19 (March 11, 2020 to September 27, 2021), and post-COVID-19 (after September 27, 2021) periods (Supplementary Table 2). These dates correspond to the World Health Organization's declaration of COVID-19 as a pandemic and the lifting of most restrictions in Sweden. In addition, logistic regression models including an interaction term between patient sex and COVID-19 period were fitted to evaluate whether the association between sex and LST-limitations differed across pandemic periods (Supplementary Table 3). To ensure robust findings, we also repeated the main analysis after excluding admissions during the COVID-19 period (Supplementary Table 4). Finally, a mixed-effects model including ICU site as a random intercept was used to account for between-ICU variation (Supplementary Table 5).

Analyses used R version 4.1.1 [11]. All tests were two-tailed and a p-value <0.05 was considered statistically significant.

## Results

The final cohort consisted of 111,354 patients from 67 ICUs after exclusion of 3,799 patients with concealed or temporary personal identity number, 1218 patients who emigrated within 30 days of admission, 2,394 patients with missing SAPS3, 13,542 patients from 13 ICUs with more than 40% missing data on LST-decisions and 10,304 patients with missing LST-decision data (Supplementary Table 6 and Fig. 1). The median age was 67 years and 41.7% were women. The overall 30-day mortality was 22.0% (Table 1).

**Fig. 1:**
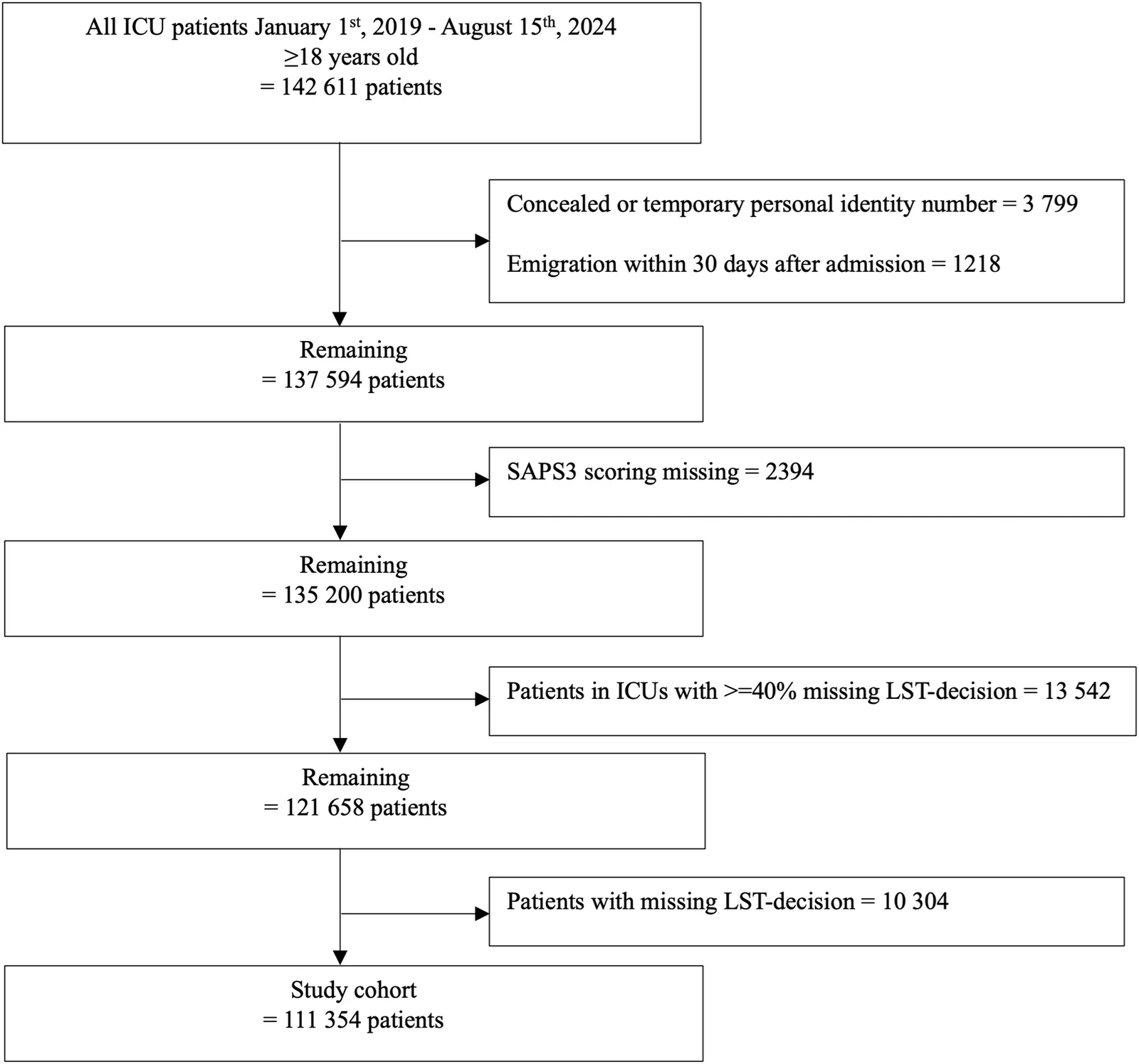
Flow chart of included patients.

**Table 1:** Patient characteristics stratified by sex.

	Women	Men	Overall
Number of patients, n (% of overall)	46426 (41.7%)	64928 (58.3%)	111354 (100%)
Age, years,median (IQR)	67 (50–77)	67 (53–76)	67 (52–76)
Age groups			
<40	7678 (16.5)	8826 (15.0)	16504 (14.8%)
40–59	9326 (20.1)	13714 (21.1)	23040 (20.7%)
60–79	21312 (45.9)	32663 (50.3)	53975 (48.5%)
≥80	8110 (17.5)	9725 (13.6)	17835 (16.0%)
SAPS3, median (IQR)	55 (44-66)	56 (45-68)	56 (45-67)
Estimated mortality risk, median (IQR)	12.2 (3.6–29.9)	13.5 (4.0–34.0)	13.5 (4.0–31.9)
**Estimated mortality groups, n (%)**			
<30%	34873 (75.1)	47014 (72.4)	81887 (73.5)
30–60%	8071 (17.4)	12236 (18.9)	20307 (18.2)
>60%	3482 (7.5)	5678 (8.7)	9160 (8.2)
**Location before ICU admission, n (%)**			
Emergency department	24146 (52.0)	33 365 (51.4)	57511 (51.6)
Hospital ward	13214 (28.5)	18 807 (29.0)	32021 (28.8)
Surgery	6064 (13.1%)	8262 (12.7%)	14326 (12.9%)
**Comorbidity in SAPS3, n (%)**			
Metastatic cancer	3677 (7.9)	5458 (8.4)	9135 (8.2)
Cancer therapy or immunosuppressive treatment^a^	2710 (5.8)	3493 (5.4)	6203 (5.6)
Chronic heart failure^b^	2514 (5.4)	4161 (6.4)	6675 (6.0)
Hematologic cancer	711 (1.5)	1241 (1.9)	1952 (1.8)
Cirrhosis	976 (2.1)	1612 (2.5)	2588 (2.3)
**Reason for admission in SAPS3, n (%)**			
Monitoring only	4475 (9.6)	5678 (8.7)	10153 (9.1)
Cardiovascular	17033 (36.7)	24416 (37.6)	41449 (37.2)
Hepatic	2118 (4.6)	2904 (4.5)	5022 (4.5)
Gastrointestinal	5186 (11.2)	7089 (10.9)	12275 (11.0)
Neurologic	16022 (34.5)	21678 (33.4)	37700 (33.9)
Renal	6977 (15.0)	10971 (16.9)	17948 (16.1)
Respiratory	15936 (34.3)	23777 (36.6)	39713 (35.7)
Hematologic	1743 (3.8)	2217 (3.4)	3960 (3.6)
Metabolic	10878 (23.4)	13628 (21.0)	24506 (22.0)
Trauma	2433 (5.2)	6364 (9.8)	8797 (7.9)
Other	5172 (11.1)	6558 (10.1)	11730 (10.5)
** 30-day mortality, n (%)**	9877 (21.3%)	14569 (22.4%)	24446 (22.0%)

IQR interquartile range, LST life-sustaining treatment, SAPS3 Simplified Acute Physiology Score 3.

a =steroid treatment >6 months, chemotherapy, immunosuppression, radiotherapy,

b =NYHA IV. Patients can be assigned several SAPS3 reasons for admission.

### LST-limitations

Table 2 describes proportions of LST-limitations in the full cohort and in subgroups. LST-limitations in the ICU were more common in women than in men (25.3% vs 23.8%, p < 0.0001). LST-limitations were more frequent in women across all EMR groups and among 30-day non-survivors.

**Table 2:** LST-limitations across EMR- and mortality groups.

	Women	Men	Overall	P-value*
**LST-limitations in all patients, n (%)**	11741 (25.3)	15447 (23.8)	27188(24.4)	**<0.0001**
**LST-limitations in EMR groups, n (%)**				
<30%	5239 (15.0)	6049 (12.9)	11288 (13.8)	**<0.0001**
30–60%	4069 (50.4)	5630 (46.0)	9699 (47.8)	**<0.0001**
>60%	2433 (69.9)	3768 (66.4)	6201 (67.7)	**0.0005**
**LST-limitations in mortality groups, n (%)**				
30-day non-survivors	7309 (74.0)	10170 (69.8)	17479 (71.5)	**<0.0001**

LST life-sustaining treatment, EMR estimated mortality risk.

* Categorical variables compared with chi-square test.

In logistic regression, presented in Table 3, the univariable model showed higher odds of LST-limitations in the ICU in women compared to men (OR 1.08, CI 1.05−1.11). This persisted after adjustment for age and SAPS3 in the multivariable model (OR 1.21, CI 1.17−1.25). Increasing age and SAPS3 were also associated with LST-limitations in the ICU in both the univariable and multivariable models. Among patients who died within 30 days, women had higher odds of receiving an LST-limitation than men (OR 1.24, CI 1.18−1.32), meaning that men who died were less likely to have had an LST-limitation in the ICU than women who died. The results were consistent when excluding the COVID-19 period (Supplementary Table 4) and when incorporating ICU site as a random intercept in the model (Supplementary Table 5). Additional analyses demonstrated that LST-limitations were more common in the post-COVID-19 period than in the pre-COVID-19 period in the overall cohort and in both sexes (Supplementary Table 2). However, the association between female sex and LST-limitations remained stable across pandemic periods, with no significant sex-by-COVID-19-period interaction (Supplementary Table 3).

**Table 3:** Univariable and multivariable logistic regression analysis for LST-limitation. In full cohort and in 30-day non-survivors. Odds ratios with corresponding 95% confidence intervals.

	n	Univariable OR (95% CI)	P-value	Multivariable* OR (95% CI)	P-value
**Full cohort**	111354				
Female sex		1.08 (1.05–1.11)	**<0.0001**	1.21 (1.17–1.25)	**<0.0001**
Age, per year		1.07 (1.07–1.08)	**<0.0001**	1.05 (1.05–1.05)	**<0.0001**
SAPS3, per 1 unit increase		1.09 (1.09–1.09)	**<0.0001**	1.07 (1.07–1.07)	**<0.0001**
**30-day non-survivors**	24446				
Female sex		1.23 (1.16–1.30)	**<0.0001**	1.24 (1.18–1.32)	**<0.0001**
Age, per year		1.03 (1.03–1.03)	**<0.0001**	1.02 (1.02–1.03)	**<0.0001**
SAPS3, per 1 unit increase		1.03 (1.03–1.04)	**<0.0001**	1.03 (1.03–1.03)	**<0.0001**

OR odds ratio, CI confidence interval, LST life-sustaining treatment.

* Multivariable adjusted for patient sex, age and SAPS3.

### Characteristics of patients with LST-limitations

Women who received LST-limitations were older and had lower SAPS3 than men (77 years vs 76, p < 0.0001; 68 vs 70, p < 0.0001) (Table 4). Among those with LST-limitations, women more often had limitations set prior to ICU admission (26.7% vs 23.9%). Median time to first LST-limitation was 3.6 h from admission. Women were assigned LST-limitations earlier than men (2.8 h vs 4.5 h, p < 0.0001). Median time from first LST-limitation to death was 7 days, with women having longer time between LST-limitation and death than men (7 days vs 6 days, p < 0.0001). Among patients who received LST-limitations, men had higher 30-day mortality than women (65.8% vs 62.3%, p < 0.0001).

**Table 4:** Characteristics of patients with LST-limitations.

	Women	Men	Overall	P-value*
Number of patients	11741	15447	27188	
Age, years, median (IQR)	77 (69–83)	76 (68–82)	76 (69–82)	**<0.0001**
SAPS3, median (IQR)	68 (60–78)	70 (61–80)	69 (61–79)	**<0.0001**
Limitation prior to the ICU, n (%)	3134 (26.7)	3689 (23.9)	6823 (25.1)	**<0.0001**
Time from admission to limitation, hours, median (IQR)	2.8 (0–21)	4.5 (0–35.5)	3.6 (0–26.5)	**<0.0001**
Time from limitation to death, days, median (IQR)	7 (1–213)	6 (1–127)	7 (1–159)	**<0.0001**
**Mortality** n (%)				
30-day mortality	7309 (62.3%)	10170 (65.8%)	17479 (64.3%)	**<0.0001**

ICU intensive care unit, IQR interquartile range, LST life-sustaining treatment.

* Continuous variables tested with Wilcoxon rank-sum test, categorical variables tested with chi-square test.

The characteristics of LST-limitations initiated in the ICU in patients without a prior limitation, are presented in Table 5. The most common documented reasons were acute disease (76.9%) and chronic disease (61.8%), followed by therapy failure (20.1%) and patient autonomy (6.7%). Acute disease and therapy failure were more often documented as reasons in men than in women (78.8% vs. 74.3%, p < 0.0001; 21.2% vs. 18.6%, p = 0.0001). In contrast, chronic disease and autonomy were more commonly documented as reasons in women than in men (63.2% vs. 60.7%, p = 0.0021; 7.8% vs. 5.9%, p < 0.0001).

**Table 5:** Characteristics of LST-limitations initiated in the ICU among patients without prior limitation before ICU admission. Time from admission to LST-limitation was available for all 20,365 patients. Variables describing reasons for LST-limitation, withholding and withdrawal of treatment, and involvement in decision-making were unavailable from 15 ICUs with complete non-reporting and were therefore analysed in 14,616 patients.

	Women	Men	Overall	P-value*
Number of patients	8607	11758	20365	
Time from admission to limitation (hours; median(IQR))	9.9 (1.8–37.4)	13.6 (2.2–61.1)	12.0 (2.0–47.5)	**<0.0001**
**Reasons**† n (%)	**n = 6281**	**n = 8335**	n **= 14616**	
Autonomy	489 (7.8%)	490 (5.9%)	979 (6.7%)	**<0.0001**
Acute disease	4665 (74.3%)	6569 (78.8%)	11234 (76.9%)	**<0.0001**
Chronic disease	3972 (63.2%)	5062 (60.7%)	9034 (61.8%)	**0.0021**
Therapy failure	1167 (18.6%)	1764 (21.2%)	2931 (20.1%)	**0.0001**
Other	149 (2.4%)	185 (2.2%)	334 (2.3%)	0.5785
**Withholding LST**† n (%)				
Invasive ventilation	2971 (47.3%)	3553 (42.6%)	6524 (44.6%)	**<0.0001**
Non-invasive ventilation	837 (13.3%)	1056 (12.7%)	1893 (13%)	0.2521
Dialysis	2474 (39.4%)	3207 (38.5%)	5681 (38.9%)	0.2700
CPR	5772 (91.9%)	7632 (91.6%)	13404 (91.7%)	0.4921
Vasoactive medication	841 (13.4%)	1091 (13.1%)	1932 (13.2%)	0.6130
Other	445 (7.1%)	679 (8.1%)	1124 (7.7%)	**0.0186**
**Withdrawing LST**† n (%)				
Invasive ventilation	743 (11.8%)	1076 (12.9%)	1819 (12.4%)	0.0532
Non-invasive ventilation	198 (3.2%)	231 (2.8%)	429 (2.9%)	0.1932
Dialysis	202 (3.2%)	291 (3.5%)	493 (3.4%)	0.3864
Vasoactive medication	737 (11.7%)	1035 (12.4%)	1772 (12.1%)	0.2194
Other	155 (2.5%)	260 (3.1%)	415 (2.8%)	**0.0216**
**In consultation with**†				
Another licensed healthcare professional	5887 (93.7%)	7831 (94%)	13718 (93.9%)	0.5970
Patient	836 (13.3%)	985 (11.8%)	1821 (12.5%)	**0.0074**
Next of kin	2161 (34.4%)	2869 (34.4%)	5030 (34.4%)	0.9982

CPR cardiopulmonary resuscitation, IQR interquartile range, LST life-sustaining treatment.

* Continious variables compared with Wilcoxon rank-sum tests, categorical variables compared with chi-square tests. Patients could be assigned several reasons for LST-limitation.

† Fifteen ICUs with complete non-reporting of variables describing reasons for LST-limitations were excluded from these analyses.

Withholding invasive ventilation was more common in women than in men (47.3% vs 42.6%, p < 0.0001), whereas withholding of “Other LSTs” was more common in men. No sex differences were observed for the remaining withholding decisions (Table 5). There were no sex differences in withdrawal of LSTs except for the category “Other LSTs”, which was more commonly withdrawn in men. Most LST-limitations (93.9%) were decided in consultation with another licensed healthcare professional. In 34.4% of cases, the next of kin were involved in the decision-making, and in 12.5%, the patient was involved. Women were involved more often in the decision-making process (13.3% vs 11.8%, p = 0.0074).

## Discussion

### Key findings

This cohort study found that women were more likely than men to receive LST-limitations, a difference that persisted after adjusting for age and SAPS3. Women also received LST-limitations earlier during their ICU stay. Among patients who died within 30 days after ICU admission, men were less likely to have had an LST-limitation in the ICU. Documented reasons for LST-limitations differed by sex: acute disease and therapy failure were more common in men, while chronic disease and autonomy were more frequent in women. Additionally, women had higher rates of documented patient involvement regarding their LST-limitations.

### Comparison with the literature

Few studies have investigated sex differences in LST-limitations in the ICU. Although most previous studies identified sex differences only as secondary findings, most support our results, showing increased LST-limitations in women both in unadjusted and adjusted analyses [7,8]. Similar patterns have been observed in diagnostic subgroups such as sepsis [12], cardiac arrest [13,14] and neurocritical care [15,16]. Some studies have reported significantly more LST-limitations in women but did not include sex in adjusted models [[17], [18], [19]]. In contrast, one study found more LST-limitations in men [20]; however, its small sample size (151 patients from a single ICU) and restriction to patients who died in the ICU limit generalisability. These conflicting findings may partly reflect that women with LST-limitations in our cohort had lower mortality than men with LST-limitations. As a result, women may be underrepresented in studies restricted to patients who die in the ICU.

A higher proportion of LST-limitations in women could represent different trajectories of critical illness between sexes, with women potentially presenting with more severe illness at ICU admission. However, the association persisted after adjustment for age and SAPS3, suggesting that women were more frequently addressed with LST-limitations even under comparable clinical conditions. There might be clinical factors, not measured in SAPS3, that vary between men and women and influence the patient’s prognosis, potentially leading to an underestimation of illness severity in women. However, prior research has consistently shown less intense treatment in women compared to men in the ICU [21], supporting our findings and contradicting the theory of underestimation of female illness severity in the ICU.

Previous research has shown an association between marital status and LST-limitations, with widowed patients being more likely to receive such limitations [22]. Furthermore, a study conducted in neurocritical care found that women receiving LST-limitations were older and more often without a partner [15]. Although marital status was not available in our dataset, widowhood is substantially more common among older women than older men in Sweden [23]. Social circumstances and partner status may therefore represent one potential explanation for the observed sex differences and should be explored in future studies.

We observed sex-based differences in documented reasons for LST-limitations. This aspect of LST-limitations is rarely studied but similar results have been shown in a smaller study including two Swedish hospitals [24]. In contrast, no difference in reasons for LST-limitations has been identified in the neurocritical care setting [15]. The higher rate of chronic disease as a documented reason for LST-limitations in women could suggest a greater burden of chronic comorbidity. However, although only a limited number of comorbidities were available in the present study, all but one were more common among men. Furthermore, a large Swedish population-based study found that men generally had slightly higher Charlson Comorbidity Index scores than women across most age groups [25]. Taken together, the available comorbidity data do not appear to account for the observed sex differences. The higher frequency of autonomy as a documented reason in women, along with greater patient involvement in their LST-limitations, may suggest sex differences in treatment preferences or discussions regarding such preferences. Women may be asked about LST preferences more often, may express their preferences more clearly, or may have a stronger inclination toward LST-limitations. A recent scoping review identified several sex and gender differences in patient-level factors related to end-of-life care, ranging from symptom experience to care preferences [26]. Prior research has shown that men in terminal illness are more reluctant than women to enter discussion regarding death and need more prompting to initiate such discussions [27], indicating that men may benefit from additional support in these decisions. Conversely, a study interviewing older patients in England found that women were twice as likely to oppose LST, often citing a desire not to burden others [28], which may reflect differing attitudes toward LST-limitations. Notably, among patients with ICU-initiated LST-limitations, invasive ventilation was more frequently withheld in women than in men, whereas no sex differences were observed for withdrawal of ongoing treatments. Few previous studies have investigated sex differences in specific LST measures, and withholding and withdrawal are often analysed together. As invasive ventilation was the only major treatment-specific limitation that differed between sexes, it may represent an important contributor to the overall sex difference in LST-limitations.

LST-limitations occurred earlier in women than in men, a difference partly explained by a higher frequency of LST-limitations prior to ICU admission in women. However, this pattern was also observed among patients without prior limitations, suggesting that sex-based differences in timing persist even among treatment-naïve individuals. In neurocritical care, earlier LST-limitations have been associated with decisions based on the patient’s own wishes, whereas later limitations are more often attributed to next-of-kin preferences [15]. A Swedish study reported that discussions regarding LST-limitations were more frequently deemed not possible in men and found that women were more often involved in the decision-making process [24]. Although patient involvement was uncommon overall in our results, the higher frequency of involvement among women may indicate that earlier decisions in women facilitate patient involvement, while the later decisions in men may occur alongside more severe clinical deterioration, limiting opportunities for direct communication. Alternatively, men may present in a condition that hinders discussion from the outset, resulting in delayed decisions and reduced incorporation of the patient’s own preferences. The higher mortality rates in men with LST‑limitations may reflect that men receive such limitations later and at a more terminal stage of illness compared with women.

Importantly, clinical practices and contextual variations surrounding the timing and approach to prognosis assessment, as well as how discussions are initiated with patients and their next of kin, will ultimately affect which patients receive LST-limitations. The COVID-19 pandemic has been hypothesised to have influenced LST-limitations by increasing awareness of end-of-life decision-making [29]. We observed higher rates of LST-limitations after the COVID-19 pandemic than before the pandemic; nevertheless, the association between female sex and LST-limitations remained consistent across pandemic periods. Additionally, physician characteristics, such as experience and attitude toward end-of-life care, may influence both the timing and the nature of these decisions. In cancer patients, female physicians have been shown to complete do-not-resuscitate (DNR) orders earlier than male physicians and to be more likely to write DNR orders for female patients [30]. However, this study had no data on physician characteristics.

### Implications

Since LST-limitations are important when intensive care risks causing more harm than good, a higher proportion of limitations is not inherently negative for women. In contrast, men’s lower rate of LST-limitations, higher SAPS3 among those with a limitation, and the finding that men who died within 30 days were less likely to have had an LST-limitation than women raise the concern that men might more often be subjected to care that is not ethically justified. Furthermore, as documented patient involvement in LST decisions was generally low and less common among men than women, efforts may be needed to ensure that discussions regarding LST-limitations are initiated for patients in the ICU, with particular attention to men. Such efforts may help promote patient-centred care and reduce unnecessary overtreatment.

### Limitations

The registry-based study design comes with certain limitations. First, treatment strategy is a relatively new registry variable, and missing data most likely reflects non-reporting rather than absence of a strategy. Patients with missing treatment strategy data had shorter ICU stays and lower mortality, suggesting that missingness may have occurred more frequently among patients without treatment limitations, potentially leading to a slight overestimation of the overall prevalence of LST-limitations. However, there was no indication that missingness in LST strategy differed between sexes, so ORs and sex comparisons should not be affected. Second, data on LST-limitation characteristics were unavailable for patients with limitations in place prior to ICU admission, restricting analyses on characteristics to decisions made in the ICU. Third, analyses of LST decision characteristics were based on a reduced cohort because 15 ICUs did not report these variables, while reporting was complete in the remaining ICUs. Consequently, these findings may not be fully representative of all participating ICUs, although the pattern suggests ICU-level reporting differences rather than missing information for individual patients. Further, the registry does not allow assessment of the completeness of documentation. Consequently, discussions, decision rationales, and patient or next-of-kin involvement that occurred but were not documented could not be captured or evaluated. Fourth, we lacked data regarding whether patient involvement was deemed possible or not. Fifth, the absence of Clinical Frailty Scale variables may affect our interpretation, as frailty, which is more common among women [31], could influence LST-limitations and potentially confound sex-based comparisons. Furthermore, we could not assess whether differences in information available to clinicians at the time of decision-making contributed to the observed sex differences in timing of LST-limitations. Lastly, studies have revealed notable differences in LST-limitations across countries and hospitals. While this nationwide study may be generalisable to other Western healthcare systems, differences in legislation, clinical practice, healthcare organisation, and societal attitudes towards end-of-life care may limit the applicability of our findings to other settings. Furthermore, Sweden is a relatively secular society, and cultural or religious factors influencing decisions regarding life-sustaining treatment may differ in other countries. In addition, information on socioeconomic, cultural, religious, and psychological factors were unavailable and may have influenced decisions regarding life-sustaining treatment.

## Conclusion

LST-limitations were more frequent and occurred earlier in women, suggesting an imbalance in end-of-life decision-making which cannot be explained by age or disease severity as documented by SAPS3 criteria. Documented reasons for LST-limitations differed by sex. While documented patient involvement was low overall, it was less common among men, who were also less likely to have had an LST-limitation among 30-day non-survivors. Efforts may be needed to ensure that discussions regarding LST-limitations are initiated for patients in the ICU, with particular attention to men.

## Authors’ contributions

EH, JE, and EL had full access to the data, ensuring its integrity, accuracy, and the decision to submit. Concept and design: EH, JE, LH, EL. Data acquisition, analysis, or interpretation: All authors. Drafting: EH, EL. Critical revision: All authors. Statistical analysis: EH, JE, EL. Administrative, technical, or material support: JE, EL. Supervision: JE, EL. All authors approved the final manuscript.

## Ethics approval

The study was approved by the Swedish Ethical Review Authority (2023-05595-01 and 2023-07554-02). The SIR operates within the legal framework of the Swedish National Quality Registries which does not require written informed consent from the patients, but patients may withdraw their data from the registry at any time.

## Consent for publication

Not applicable.

## Declaration of Generative AI and AI-assisted technologies in the writing process

During the preparation of this work the authors used Microsoft Copilot in order to review the text and improve its readability. After using this tool, the authors reviewed and edited the content as needed and take full responsibility for the content of the publication.

## Funding

Funding was provided by 10.13039/501100004047Karolinska Institutet and Region Stockholm. They had no role in the study's design, conduct, data analysis, or manuscript preparation and submission.

## Data availability

The dataset is not publicly available, as it contains personal data that the ethical permission does not allow to share. On reasonable request data could be made available after permission from the Swedish Ethical Review Authority and SIR.

## Declaration of competing interest

The authors declare that they have no competing interests.
