# Supplementary material for: Sex differences in limitations of life-sustaining treatment in the intensive care unit: A nationwide cohort study
Source: Ann Intensive Care. 2026 Oct 17;16:100190. doi: 10.1016/j.aicoj.2026.100190 (PMC13740874; doi:10.1016/j.aicoj.2026.100190)
Supplement: Supplementary file 1 [file mmc1.docx]

**SUPPLEMENTARY MATERIAL**

**PAPER III**

**Sex Differences in Limitations of Life-sustaining Treatment in the Intensive Care Unit: A Nationwide Cohort Study**

Elsa Hägglöf, Jesper Eriksson, Max Bell, Linn Hallqvist, Lars Engerström and Emma Larsson

Table of Contents

[Supplementary Table 1.	2](#_Toc242621306)

[Supplementary Table 2.	2](#_Toc242621307)

[Supplementary Table 3.	3](#_Toc242621308)

[Supplementary Table 4.	3](#_Toc242621309)

[Supplementary Table 5.	4](#_Toc242621310)

[Supplementary Table 6.	5](#_Toc242621311)

# **Supplementary Table 1.**

**Sensitivity analysis of ICU and patient exclusion according to different thresholds for missing treatment strategy documentation.** Number of ICUs and patients excluded when applying different thresholds for the proportion of patients with missing treatment strategy data. The primary analysis used a threshold of >40% missing data, excluding 13 ICUs and 13,542 patients.

| **Missingness threshold** | **Excluded ICUs** | **Excluded patients** |
| --- | --- | --- |
| >20% | 23 | 26338 |
| >30% | 17 | 21715 |
| >40% | 13 | 13542 |
| >50% | 10 | 7268 |
| >60% | 8 | 4963 |

# **Supplementary Table 2.**

**LST-limitations before, during, and after the COVID-19 pandemic.** Values are presented as n/N (%). The proportion of patients with documented LST-limitations overall and stratified by sex across the Pre-COVID, COVID, and Post-COVID periods. P-values in each row compare women and men within the respective COVID period, whereas p-values in the bottom row compare LST-limitation rates across COVID periods within women, within men, and in the overall cohort. Chi-square tests were used for all comparisons.

|  | **LST-limitations n/N (%)** | | |  | | |
| --- | --- | --- | --- | --- | --- | --- |
| **COVID period^a^** | **Women** | **Men** | **Overall** | **P-value female vs male** | | |
| Pre-COVID | 2742/11322 (24.2) | 3427/14955 (22.9) | 6169/26277 (23.5) | **0.0142** | | |
| COVID | 2954/12645 (23.4) | 4345/19591 (22.2) | 7299/32236 (22.6) | **0.0138** | | |
| Post-COVID | 6045/22459 (26.9) | 7675/30382 (25.3) | 13720/52841 (26.0) | **<0.0001** | | |
| **P-value across periods** | **<0.0001** | **<0.0001** | **<0.0001** |  |  |  |

a, COVID period was categorized as Pre-COVID (before 11 March 2020), COVID (11 March 2020 to 27 September 2021), and Post-COVID (after 27 September 2021)

# **Supplementary Table 3.**

**Univariable and multivariable logistic regression analysis for LST-limitation including COVID-19 period interaction.** Univariable and multivariable logistic regression analyses of factors associated with limitation of life-sustaining treatment (LST) in the full cohort. Odds ratios with corresponding 95% confidence intervals.

|  | **n** | **Univariable**  **OR (95% CI)** | **P-value** | **Multivariable***  **OR (95% CI)** | **P-value** |
| --- | --- | --- | --- | --- | --- |
| **Full cohort** | 111354 |  |  |  |  |
| Female sex |  | 1.08 (1.05–1.12) | **<0.0001** | 1.22 (1.14–1.31) | **<0.0001** |
| Age, per year |  | 1.07 (1.07–1.07) | **<0.0001** | 1.05 (1.05–1.05) | **<0.0001** |
| SAPS3, per 1 unit increase |  | 1.09 (1.09–1.09) | **<0.0001** | 1.07 (1.07–1.07) | **<0.0001** |
| COVID period^a^ |  |  |  |  |  |
| Pre-COVID |  | ref |  | ref |  |
| COVID |  | 0.95 (0.92–0.99) | **0.0171** | 1.09 (1.02–1.15) | **0.008** |
| Post-COVID |  | 1.14 (1.10–1.18) | **<0.0001** | 1.08 (1.02–1.14) | **0.009** |
| Interaction term |  |  |  |  |  |
| Female sex x Pre-COVID |  | ref |  | ref |  |
| Female sex x COVID |  | 0.99 (0.92–1.08) | 0.8986 | 0.95 (0.86–1.04) | 0.2469 |
| Female sex x Post-COVID |  | 1.01 (0.95–1.09) | 0.7047 | 1.01 (0.93–1.10) | 0.7351 |
|  |  |  |  |  |  |

OR odds ratio, CI confidence interval, LST life-sustaining treatment. a, COVID period was categorized as Pre-COVID (before 11 March 2020), COVID (11 March 2020 to 27 September 2021), and Post-COVID (after 27 September 2021), with Pre-COVID used as the reference category. Interaction terms between patient sex and COVID period were included to assess whether the association between sex and LST-limitation differed across pandemic periods. Men and pre-COVID period as reference. *Multivariable adjusted for patient sex, age, SAPS3, COVID-period and including an interaction term between patient sex and COVID-period.

# **Supplementary Table 4.**

**Univariable and multivariable logistic regression analyses for LST-limitation, excluding patients admitted 2020-03-11 to 2021-09-27 (COVID-19 period).** In full cohort and in 30-day non-survivors. Odds ratios with corresponding 95% confidence intervals.

|  | **n** | **Univariable**  **OR (95% CI)** | **P-value** | **Multivariable***  **OR (95% CI)** | **P-value** |
| --- | --- | --- | --- | --- | --- |
| **Full cohort** | 79118 |  |  |  |  |
| Female sex |  | 1.08 (1.05–1.12) | **<0.0001** | 1.23 (1.18–1.28) | **<0.0001** |
| Age, per year |  | 1.07 (1.07–1.07) | **<0.0001** | 1.05 (1.05–1.05) | **<0.0001** |
| SAPS3, per 1 unit increase |  | 1.09 (1.09–1.09) | **<0.0001** | 1.07 (1.07–1.08) | **<0.0001** |
| **30-day non-survivors** | 17323 |  |  |  |  |
| Female sex |  | 1.19 (1.12–1.28) | **<0.0001** | 1.22 (1.14–1.32) | **<0.0001** |
| Age, per year |  | 1.03 (1.03–1.03) | **<0.0001** | 1.02 (1.02–1.03) | **<0.0001** |
| SAPS3, per 1 unit increase |  | 1.03 (1.03–1.04) | **<0.0001** | 1.03 (1.03–1.03) | **<0.0001** |
|  |  |  |  |  |  |

CI confidence interval, LST life-sustaining treatment. *Multivariable adjusted for patient sex, age and SAPS3.

# **Supplementary Table 5.**

**Univariable and multivariable mixed-effects logistic regression analyses for LST-limitation, with county included as random effect.** In full cohort and in 30-day non-survivors. Odds ratios with corresponding 95% confidence intervals.

|  | **n** | **Univariable**  **OR (95% CI)** | **P-value** | **Multivariable***  **OR (95% CI)** | **P-value** |
| --- | --- | --- | --- | --- | --- |
| **Full cohort** | 111354 |  |  |  |  |
| Female sex |  | 1.07 (1.04–1.10) | **<0.0001** | 1.20 (1.16–1.24) | **<0.0001** |
| Age, per year |  | 1.08 (1.07–1.08) | **<0.0001** | 1.05 (1.05–1.05) | **<0.0001** |
| SAPS3, per 1 unit increase |  | 1.09 (1.09–1.09) | **<0.0001** | 1.07 (1.07–1.08) | **<0.0001** |
| **30-day non-survivors** | 24446 |  |  |  |  |
| Female sex |  | 1.22 (1.15–1.30) | **<0.0001** | 1.24 (1.17–1.31) | **<0.0001** |
| Age, per year |  | 1.03 (1.03–1.03) | **<0.0001** | 1.03 (1.02–1.03) | **<0.0001** |
| SAPS3, per 1 unit increase |  | 1.04 (1.03–1.04) | **<0.0001** | 1.03 (1.03–1.03) | **<0.0001** |
|  |  |  |  |  |  |

OR odds ratio, CI confidence interval, LST life-sustaining treatment. *Multivariable adjusted for patient sex, age and SAPS3, county as random effect.

# **Supplementary Table 6.**

**Patient characteristics among patients with missing versus documented LST-decisions**

|  | **Patients with missing LST-decisions** | **Patients with documented LST-decisions** |
| --- | --- | --- |
| Number of patients, no. | 10304 | 111354 |
| Age, years, median (IQR) | 66 (51–76) | 67 (52–76) |
| Age groups, no. (%) |  |  |
| <40 | 1641 (15.9%) | 16504 (14.8%) |
| 40-59 | 2239 (21.7%) | 23040 (20.7%) |
| 60-79 | 4928 (47.8%) | 53975 (48.5%) |
| ≥80 | 1496 (14.5%) | 17835 (16.0%) |
| Female sex, no. (%) | 4408 (42.8%) | 46426 (41.7%) |
| SAPS3, median (IQR) | 52.0 (42–63) | 56.0 (45–67) |
| Estimated mortality risk, median (IQR) | 9.0 (2.7–24.2) | 13.5 (4.0–31.9) |
| LOS, hours, median (IQR) | 19.9 (8.5–42.4) | 28.2 (14.2–70.8) |
| **Estimated mortality groups, no. (%)** |  |  |
| <30% | 8371 (81.2%) | 81887 (73.5%) |
| 30-60% | 1432 (13.9%) | 20307 (18.2%) |
| >60% | 501 (4.9%) | 9160 (8.2%) |
| **Location before ICU admission, no. (%)** |  |  |
| Emergency department | 5319 (51.6%) | 57511 (51.6%) |
| Hospital ward | 2428 (23.6%) | 32021 (28.8%) |
| **Comorbidity in SAPS3, no. (%)** |  |  |
| Metastatic cancer | 967 (9.4%) | 9135 (8.2%) |
| Cancer therapy or immunosuppressive treatment^a^ | 415 (4.0%) | 6203 (5.6%) |
| Chronic heart failure^b^ | 495 (4.8%) | 6675 (6.0%) |
| Hematologic cancer | 139 (1.3%) | 1952 (1.8%) |
| Cirrhosis | 179 (1.7%) | 2588 (2.3%) |
| **Reason for admission in SAPS3, no. (%)** |  |  |
| Observation only | 1906 (18.5%) | 10153 (9.1%) |
| Cardiovascular | 3303 (32.1%) | 41449 (37.2%) |
| Hepatic | 356 (3.5%) | 5022 (4.5%) |
| Gastrointestinal | 998 (9.7%) | 12275 (11.0%) |
| Neurologic | 3285 (31.9%) | 37700 (33.9%) |
| Renal | 1300 (12.6%) | 17948 (16.1%) |
| Respiratory | 2902 (28.2%) | 39713 (35.7%) |
| Hematologic | 301 (2.9%) | 3960 (3.6%) |
| Metabolic | 1986 (19.3%) | 24506 (22.0%) |
| Trauma | 846 (8.2%) | 8797 (7.9%) |
| Other | 1129 (11.0%) | 11730 (10.5%) |
| **30-day mortality, no. (%)** | 1328 (12.9%) | 24446 (22.0%) |

IQR interquartile range, LST life-sustaining treatment, SAPS3 Simplified Acute Physiology Score 3. a = steroid treatment >6 months, chemotherapy, immunosuppression, radiotherapy, b=NYHA IV. Patients can be assigned several SAPS3 reasons for admission
